# Amikacin Liposomal Inhalation Suspension for Non-Tuberculous *Mycobacteria* Lung Infection: A Greek Observational Study

**DOI:** 10.3390/medicina60101620

**Published:** 2024-10-03

**Authors:** Angeliki A. Loukeri, Evgenia Papathanassiou, Aikaterini Kavvada, Christos F. Kampolis, Ioannis Pantazopoulos, Charalambos Moschos, Apostolos Papavasileiou

**Affiliations:** 1 Department of Mycobacterial Diseases, “Sotiria” Hospital, 11527 Athens, Greece; angelouk@live.com (A.A.L.); evgjenaki11@yahoo.com (E.P.); katerinakavvada@yahoo.com (A.K.); hamosgr@hotmail.com (C.M.); papgrower@gmail.com (A.P.); 2 Department of Emergency Medicine, “Hippokration” General Hospital of Athens, 11527 Athens, Greece; chkamp77@gmail.com; 3 Department of Respiratory Medicine, Faculty of Medicine, University of Thessaly, Mezourlo, 41110 Larissa, Greece

**Keywords:** mycobacterial infections, *Mycobacterium avium* complex, *Mycobacterium abscessus*, aminoglycoside, liposome, inhalation

## Abstract

*Background and Objectives:* Intravenous amikacin, recommended for severe or recurrent *M. avium complex* (MAC) infections and as initial treatment for *M. abscessus* lung disease, is often limited by serious adverse effects such as renal and auditory toxicities. Inhaled Amikacin Liposome Inhalation Suspension (ALIS) enhances pulmonary drug deposition while minimizing systemic adverse effects, and it has recently been introduced as an add-on therapy for refractory MAC infections or when other standard treatments are inadequate. This study aims to retrospectively describe the outcomes of Greek patients with difficult-to-treat non-tuberculous mycobacterial (NTM) lung disease following the addition of ALIS to guideline-based therapy. *Materials and Methods:* Seventeen consecutive patients (median age: 66 years) treated with ALIS as an add-on therapy to a standard regimen at “Sotiria” General Hospital of Chest Diseases (Athens, Greece) from 2020 to 2023 were enrolled in this study. These patients had recurrent or refractory NTM lung disease and/or limited treatment options due to prior treatment-related adverse effects. Clinical, radiological, and microbiological data on treatment response and overall outcomes after ALIS initiation were recorded for each patient. *Results:* By the end of 2023, 14 out of 17 patients had either successfully completed or were continuing their ALIS therapy. At 6 months, 85.7% (12/14) showed clinical, microbiological, and radiological improvement. However, 25% (3/12) of treated patients, primarily those with monomicrobial or combined *M. abscessus* lung disease, experienced disease relapse after therapy completion. The most frequent adverse effects related to ALIS were mild and localized to the respiratory tract, with only one patient discontinuing therapy due to hypersensitivity pneumonitis. *Conclusions:* Adding ALIS to standard regimens was effective and safe in a small group of Greek patients with refractory or recurrent NTM lung disease, particularly those who had discontinued intravenous aminoglycosides due to significant adverse effects, with notable responses observed in MAC lung disease. Further research is needed to validate these findings in clinical practice and to investigate ALIS’s role in NTM lung disease caused by other species.

## 1. Background

Non-tuberculous mycobacteria are widespread in the environment and include over 200 species and subspecies, each with varying ability to cause lung disease. The global incidence of non-tuberculous mycobacterial (NTM) lung disease seems to be increasing, particularly within specific regions and demographic groups around the world [1]. This rise could be attributed to various factors such as longer life expectancy, increased burden of chronic diseases, intensification of immunosuppressive therapies, increased availability and advances in mycobacterial diagnostic techniques, and increased awareness of NTM lung disease (NTM-LD) [2]. The growing number of patients, different NTM species and sensitivity patterns, existing co-morbidities, and likely side effects of established therapies for NTM-LD underscore the necessity for expanding our therapeutic armamentarium with novel, effective, and safe drugs.

Current guideline-based therapy for treating MAC pulmonary disease primarily recommends macrolide-based combination regimens. Intravenous (i.v.) amikacin is currently recommended for severe or recurrent *Mycobacterium avium* (*M. avium* or MAC) complex infections and as a component of the initial phase treatment for *M. abscessus* pulmonary disease [3]. However, even with these treatment regimens, less than 70% of patients with MAC pulmonary disease achieve successful outcomes, and recurrences are common [4], indicating that a substantial portion of patients continue to struggle with a chronic, ongoing disease [5,6]. Moreover, use of i.v. aminoglycosides is often restricted due to the associated risk of systemic serious adverse effects (AEs), particularly renal and auditory toxicities.

Amikacin Liposome Inhalation Suspension (ALIS; Arikayce^®^) is a liposomal formulation of amikacin administered through inhalation. ALIS consists of small in size (~300 nm in diameter), mostly unilamellar, neutrally charged, and highly biocompatible liposomes. These liposomes have been proven to be stable and resistant to the high sheer forces encountered during delivery through the vibrating mesh Pari e-flow nebulizer (Pari GmbH, Germany) [7]. They are composed of the natural lipids dipalmitoylphosphatidylcholine (DPPC) and cholesterol that encapsulate amikacin with a high amikacin-to-lipid mass ratio (approximately 1.4). This high drug-to-lipid ratio provides efficient drug delivery and achieves the recommended dose (70 mg/mL amikacin base) in patients with MAC lung disease [8,9].

DPPC, the primary lipid component, has a high melting temperature (~41 °C), which means it is in a gel state at body temperature, making the liposomal membrane more rigid. The inclusion of cholesterol at intermediate concentrations (20–30%), as seen in ALIS, modulates membrane fluidity, reducing it at high temperatures and increasing it at low temperatures [10]. Thus, instead of a clear transition between rigid and fluid states, cholesterol broadens the phase transition, allowing the liposome membrane to maintain fluidity and stability over a wider temperature range and ensuring the structural integrity of the liposomes during nebulization and delivery. The stability of these liposomes is crucial for providing consistent drug delivery over time, as they preserve their structure and keep amikacin encapsulated [9]. In terms of cellular uptake, there are four possible mechanisms for liposome-cell interactions: (a) specific interactions with cell-surface receptors or constituents, (b) internalization by phagocytic cells (endocytosis), (c) fusion of liposomal lipids into the cell membrane, and (d) lipid exchange with the cell membrane [11]. Given the gel-phase nature of DPPC at 37 °C, which results in a more rigid membrane structure and reduces the likelihood of fusion with cellular membranes, endocytosis is considered to be the primary pathway for cellular uptake of ALIS liposomes. On the other hand, high cholesterol concentrations in liposomes typically inhibit fusion, but intermediate concentrations might still allow for some degree of membrane fusion.

The drug is inhaled by the patient via the oral route and subsequently taken up by alveolar macrophages, which represent an intracellular niche where NTM can reside, allowing for targeted action against the bacteria [9]. Compared to inhaled free amikacin, ALIS inhalation is associated with a ~4-fold increased amikacin uptake into cultured macrophages [12]. Additionally, when ALIS is administered via nebulization and inhalation, it is delivered straight to the lungs, leading to a much higher concentration of amikacin in the sputum than in the bloodstream after the dose is given. Thus, inhaled ALIS results in markedly lower systemic concentrations of the drug compared to the intravenous administration of amikacin sulfate [13]. In this manner, ALIS facilitates localized drug delivery to the lungs, thereby mitigating systemic exposure and minimizing potential AEs. 

The addition of ALIS was proven to be effective in patients not responding to standard guideline-based treatment for MAC-associated lung disease (MAC-LD) by increasing sputum culture conversion rates [14,15].In the CONVERT study, culture conversion was achieved in 65 out of 224 patients (29%) receiving ALIS plus guideline-based therapy, compared to 10 out of 112 patients (8.9%) receiving guideline-based therapy alone. No significant differences were observed between the treatment arms regarding patient-reported outcomes on the St. George’s Respiratory Questionnaire [14]. Later, in September 2023, interim results released from the Phase 3 ARISE study showed positive outcomes, such as higher culture conversion rates and improved quality of life, as validated using the QOL-B questionnaire, in patients with newly diagnosed MAC lung disease [16]. Currently, ALIS has received U.S. FDA approval as part of a combination of antibacterial drug regimens for patients with refractory MAC-LD (i.e., persistent active infection despite six consecutive months of the standard guideline-based regimen) and in Europe for patients with limited or no alternative treatment options.

This retrospective case series aims to present the effect of ALIS as an add-on therapy to existing multidrug regimens, on the outcome of patients with treatment-naive, refractory, or recurrent MAC- or *M. abscessus*-LD in a real-world clinical setting.

## 2. Materials and Methods

### 2.1. Study Population

This study was conducted on a selected cohort of patients treated with ALIS for NTM-LD from 2020 to 2023 at the Department of Mycobacterial Diseases of “Sotiria” General Hospital of Chest Diseases (Athens, Greece). Patients aged > 18 years with recurrent or refractory NTM-LD and/or limited treatment options due to standard treatment-related AEs were eligible for treatment with ALIS. Patients were included if they met the clinical and microbiological diagnostic criteria for NTM (*M. avium*, *M. intracellulare*, *M. abscessus*) lung disease as outlined by the American Thoracic Society and the Infectious Diseases Society of America (ATS/IDSA) guidelines. Specifically, inclusion required the following: (i) the presence of respiratory symptoms consistent with NTM lung disease accompanied by multifocal bronchiectasis and numerous nodules on chest CT scans, (ii) the exclusion of alternative causes, and (iii) confirmation of the laboratory diagnosis through positive cultures from either two sputum samples or one bronchoalveolar lavage or washing sample [17]. All patients had received a minimum of 4 weeks of treatment with ALIS as add-on therapy to guideline-based treatment. Patients with extrapulmonary or disseminated disease and/or cystic fibrosis (CF) were excluded. The following information was recorded for all patients: demographics (sex, age, body mass index (BMI), smoking habit), co-morbidities, concomitant medications, symptoms prior to diagnosis and at treatment initiation (e.g., cough, sputum production, dyspnea, hemoptysis, fever), and other antibiotic use prior to or in parallel with ALIS administration. 

### 2.2. Laboratory and Radiological Examinations

Microbiological assessments of sputum or bronchoscopic specimens were performed at the Mycobacteria Department of “Sotiria” Hospital, which is the nationally recognized reference laboratory. In addition, bronchoscopy was performed either 6 months after the initiation of ALIS, or sooner in cases of clinical and/or radiological deterioration. Similarly, all patients underwent a CT scan both before starting ALIS and after 6 months, or earlier if there was evidence of clinical worsening. This approach ensured the comprehensive monitoring of both microbiological and radiological parameters throughout the treatment. 

### 2.3. ALIS Administration

ALIS was administered to all patients once daily in single-use vials at a concentration of 590 mg/8.4 mL (or 70 mg/mL) [14,15] via continuous oral inhalation using the Lamira Nebulizer System, a product-specific nebulizer that has been optimized for ALIS’s formulation administration. ALIS nebulization with Lamira Nebulizer delivers both liposome-encapsulated amikacin (≥70%) and free amikacin (≥30%) via aerosol droplets of a mass median aerodynamic diameter within the respirable range (<5 μm), thus enabling acceptable drug distribution in the lungs [8]. ALIS vials can be safely stored at temperatures between 2 °C and 8 °C until their expiration date. Once at a room temperature lower than 25 °C, it can be stored for no more than 4 weeks. Adverse events associated with both standard and ALIS treatments were systematically recorded. Clinical and laboratory evaluations, including assessments of renal function, were conducted monthly to monitor patient health and treatment effects. Ototoxicity and vestibular toxicity were assessed by an otorhinolaryngologist prior to the initiation of therapy and subsequently every 2–3 months, in accordance with local clinical guidelines. Additionally, overall treatment duration, patient outcomes, and functional status were thoroughly documented and reported to assess the efficacy and safety of the ALIS regimen.

### 2.4. Statistics

Summary statistics for quantitative markers were expressed as medians and interquartile ranges, while percentages were used for qualitative markers. 

## 3. Results

### 3.1. Baseline Clinical and Laboratory Characteristics

Seventeen patients [median age 66 years (range: 55–71)] fulfilled all of the inclusion criteria, with almost two thirds (n = 11) being female. The majority of them had a history of bronchiectasis and frequent lower tract respiratory infections (94.1% and 82.3%, respectively). At the diagnosis of NTM-LD, cough was the predominant symptom (94.1%), followed by hemoptysis and weight loss (35.3% each). Bronchiectases, nodules, and extensive (>2 lobes involved) disease were the main radiological findings in almost all patients. A single mycobacterial pathogen—*M. avium*, *M. intracellulare*, and *M. abscessus* (subsp. *abscessus*), in order of decreasing frequency—was isolated as the causative agent in more than 75% of NTM-LD patients; guideline-based standard treatment was decided according to the isolated NTM species, which in most cases were sensitive to macrolides and amikacin (Table 1).

### 3.2. Treatment Prior to ALIS

Thirteen patients (76.5%) had received parenteral amikacin thrice a week for a median period of 6 months as part of the initial regimen prior to ALIS initiation, either due to the extensive nature of the disease or because of AEs from other antimicrobial agents (Table 2). The most common AEs of the initial regimens included ototoxicity (loss of hearing or tinnitus) (29.4%), nephrotoxicity (17.6%), and decreased hemoglobin levels (11.8%).

### 3.3. ALIS Add-On Therapy and Patient Outcome

The decision to add ALIS to standard regimens was made primarily due to AEs of the initial therapy and secondarily due to refractory or recurrent disease. Among the 17 patients receiving ALIS, 12 successfully completed their regimen, while 2 have been on ALIS for over six months but have not yet completed treatment. The other three discontinued their therapy due to clinically significant hypersensitivity pneumonitis, patient preference, or death from causes unrelated to NTM-LD. Three patients received both parenteral and inhaled amikacin simultaneously for a median duration of two months.

**Figure 1:**
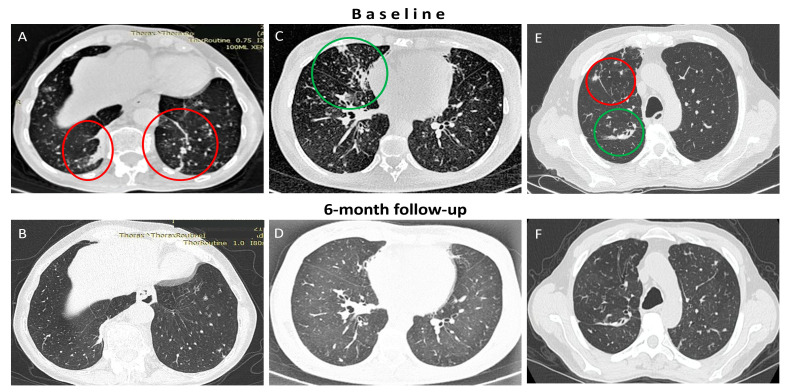
**Radiological improvement of patients on treatment with ALIS for NTM-LD.** Six months after ALIS initiation, all patients with MAC-associated LD (Panels (**A**–**D**)) and some patients with *M. abscessus-LD* (Panels (**E**,**F**) presented an improvement in CT findings such as nodules (red circles) and consolidations (green circles). (*ALIS: Amikacin liposomal inhalation suspension, MAC: M. avium complex, NTM: non-tuberculous mycobacteria, LD: lung disease*).

Among the 14 patients who received ALIS for at least 6 months, all of them manifested clinical improvement after the first 3 months, while most (12/14) also showed improvement in CT-scan findings at the 6-month reassessment (Figure 1). 

**Figure 2:**
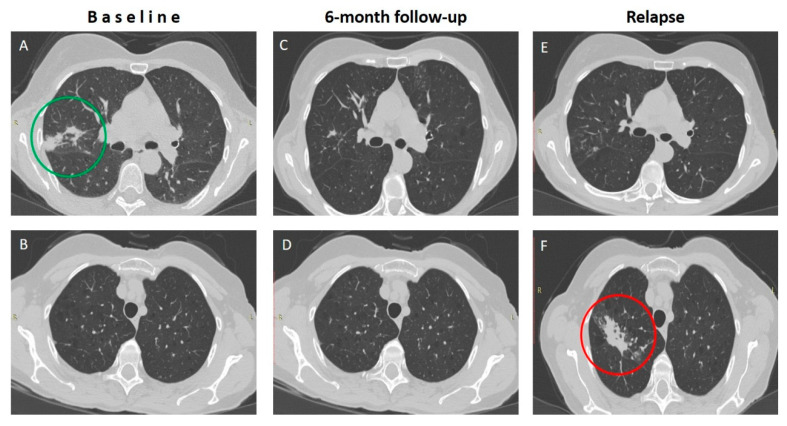
**Radiological relapse in a patient with *M. abscessus* LD during treatment with ALIS.** A patient with *M. abscessus*-associated LD showed remarkable clinical and radiological improvement (green circle) after 6 months on treatment with ALIS (Panels (**A**–**D**)). However, a few months later, he had symptom recurrence and a new infiltrate (red circle) at a different segment of the right upper lobe (Panels (**E**,**F**)). (*LD: lung disease*).

After the introduction of ALIS, all patients showed negative cultures at the 6-month mark. The median duration of ALIS treatment was 13 months. While all seven patients with MAC-LD that received ALIS for at least 6 months remained stable in their clinical, radiological, and microbiological status, two out of three patients with monomicrobial *M. abscessus*-LD experienced relapses, presenting with new positive cultures as well as radiological and clinical deterioration (Figure 2). A similar outcome was observed in one out of four patients with a combined infection. Finally, 75% (9/12) of patients completing ALIS treatment achieved sustained clinical and laboratory responses, while the rest experienced disease relapse after completing therapy.

### 3.4. ALIS-Associated Adverse Events

The most commonly reported adverse events associated with ALIS administration were localized to the respiratory tract, affecting both the upper and lower airways. These events were predominantly mild in nature. Hoarseness and dysphonia were reported by approximately one-third of patients, while bronchospasm was observed in four patients and was effectively managed with inhaled bronchodilators. Importantly, in all cases, the adverse events were mild enough to be managed with symptomatic treatment, and none of the patients had to discontinue the inhaled antibiotic therapy due to these side effects.

## 4. Discussion

This is the first observational study describing the outcome of Greek patients with NTM-LD treated with ALIS as add-on therapy to standard regimens. This treatment was administered either due to refractory or recurrent disease or, most significantly, due to serious AEs that did not permit the continuation of previous guideline-based treatment [18,19]. Our data, showing that the majority of these patients responded satisfactorily to this novel therapy, are quite encouraging for the future management of this difficult-to-handle group of patients. It is also of note that systemic AEs were minimized following the implementation of ALIS and the restriction of concomitant intravenous aminoglycosides, providing initial evidence for the more favorable safety profile of this new antibiotic formulation, which is associated primarily with local and manageable AEs.

One of the main challenges faced by clinicians when treating patients with NTM-LD are the low success rates of standard therapy even after completing treatment, with success rates as low as 65% for MAC and 45.6% for *M. abscessus* infections [6,20,21]. Moreover, high antibiotic doses directly deposited to the lungs are required in order to minimize systemic exposure, thus avoiding systemic AEs. Administration of antimicrobial agents through inhalation represents a significant advancement in this area. In the CONVERT study, a randomized Phase 3 study conducted across 18 countries, the efficacy and safety of ALIS was evaluated as an add-on treatment to standard guideline-based therapy in patients with refractory MAC-LD. In line with our findings, culture conversion by month 6 was achieved in significantly more patients compared to those receiving standard treatment alone. This effect proved to be durable since a significantly higher number of patients remained culture-negative at both three and twelve months after treatment [14,22]. In our study, introduction of ALIS was associated with clinical improvement in 14 out of 17 patients, regarding respiratory and systemic symptoms (such as cough, fatigue and loss of weight) within a median time of only three months. The median duration of ALIS regimen use was 13 months (range: 7.5 to 21.5), slightly longer than reported in other real-world studies [23,24].

ALIS has not yet been officially approved for *M. abscessus* disease. Nevertheless, in some expert centers, it is used as an off-label therapy aimed at improving outcomes for these patients, who have a condition that is inherently difficult to treat [23,24]. Existing observational data are promising; however, determining the additive effect of ALIS remains challenging. The recent NTM-NET study, which included a relatively large cohort of patients with *M. abscessus*-LD (n = 41), reported a favorable outcome in 61% of patients. However, a notable proportion still experienced treatment failure or death (31.7% and 4.9%, respectively) [24]. In our study, one patient with *M. abscessus*-LD and two patients with combined infections that included *M. abscessus* as a causative agent successfully completed treatment with ALIS and remained stable six months after finishing treatment. It is important to note that all three patients who exhibited deterioration had *M. abscessus*-LD. In two of them, *M. abscessus* was the sole causative agent, while in the third patient, it was present alongside MAC. 

Despite the efficacy and sustained treatment effect of ALIS on NTM-LD, some local AEs should be taken into consideration. In the CONVERT study, the most common AEs originated from the respiratory tract and occurred in approximately 87% of patients. Most of them were mild to moderately severe and were mainly reported soon after ALIS introduction. Fortunately, in most cases, these AEs did not result in treatment discontinuation, and systemic exposure-related AEs associated with amikacin were uncommon [14]. Real-life data reinforce these findings, as AEs reported in 81.8% of treated patients were usually mild, non-systemic, and primarily affected the respiratory tract (hoarseness, dysphonia, bronchospasm, increased cough and dyspnea) [23,25,26]. In agreement with the current literature, none of our patients discontinued treatment due to significant AEs, with the exception of one person suffering from hypersensitivity pneumonitis. The initiation of bronchodilators or short-term ALIS interruptions was part of symptomatic management for patients experiencing local respiratory AEs. Although infrequently reported, ototoxicity due to ALIS is often mild or even reversible [14,24,25]. In our study, only one patient experienced worsening of hearing impairment, which had been previously established at a certain level due to the prior parenteral use of amikacin.

The morbidity burden associated with NTM-LD and systemic treatment AEs is high, particularly in refractory or recurrent cases. The addition of ALIS to standard regimens seems to decrease health care utilization and hospitalization rates [27]. This trend was also evident in our study; despite the small sample size, only four patients were hospitalized during ALIS treatment, including those considered non-responders and one who developed hypersensitivity pneumonitis.

### Limitations and Strengths of the Study

Despite the valuable insights this study provides into the real-world use of ALIS for NTM lung disease, several limitations should be acknowledged. First, as a consequence of the relatively recent introduction of ALIS into clinical practice, this study managed to recruit a relatively small number of patients, and in most cases, follow-up time was limited to less than 24 months. Moreover, the retrospective design and heterogeneity of patient characteristics at the time of inclusion (e.g., NTM species variability, disease duration, and previous use of antimicrobial agents before ALIS initiation) were among the weaknesses of our research. Additionally, the small patient population may restrict the ability to draw definitive conclusions regarding the effectiveness of ALIS across different patient characteristics, such as age, gender, and comorbidities. As a result, subgroup analyses may lack sufficient statistical power to provide meaningful insights.

Nonetheless, this study possesses notable strengths that contribute to its significance in understanding the treatment of NTM-LD with ALIS. Firstly, it is the first observational study focusing specifically on Greek patients, providing valuable insights into a population that has been underrepresented in the literature. Secondly, the real-world setting allows for the evaluation of ALIS in a diverse cohort with varying clinical profiles, enhancing the generalizability of the findings. Additionally, this study highlights promising outcomes associated with ALIS, including clinical, radiological, and microbiological improvements, particularly in patients with refractory or recurrent NTM-LD. The comprehensive monitoring of adverse events and the emphasis on patient-reported outcomes further strengthen this study’s reliability. This detailed examination of ALIS’s application reflects the complexities and challenges associated with managing NTM lung disease. The inclusion of diverse patient profiles enhances the understanding of ALIS’s efficacy and safety across various scenarios. Furthermore, the findings contribute to the growing body of evidence supporting ALIS as a viable therapeutic option, paving the way for future research and improved management strategies for this challenging condition, underscoring the considerable efforts made by expert centers to provide the most current and comprehensive data.

## 5. Conclusions

ALIS administration as an add-on therapy to standard regimens appeared effective and safe in a small cohort of Greek patients with refractory or recurrent NTM-LD, particularly those who had to discontinue intravenous aminoglycosides due to serious AEs. Clinical, radiological, and microbiological responses were specifically remarkable in MAC-LD. 

The observed effects of ALIS in treating NTM-LD, especially MAC-LD, are attributed to its targeted drug delivery and unique pharmacological properties. ALIS’s effectiveness likely arises from its ability to deliver concentrated, prolonged drug action within the lungs while minimizing systemic side effects, thus allowing patients to continue treatment for longer periods, even those who have had to discontinue systemic aminoglycoside therapies due to serious side effects. However, future randomized controlled trials involving larger populations are necessary to extrapolate our findings to daily clinical practice, to assess the long-term prognosis of these patients, and to investigate the role of ALIS in NTM-LD caused by species other than MAC. 

## Figures and Tables

**Table 1:** Baseline characteristics of patients with NTM-LD (*n* = 17).

**Characteristic**	**Median (Range)**
Age	66 (55–71)
**Characteristic**	**No. of Patients (%)**
**Sex**	
Female	11 (64.7%)
**Past Medical History**	
Bronchiectasis	16 (94.1%)
Frequent LTRIs (Lower Tract Respiratory Infections)	14 (82.3%)
COPD (Chronic Obstructive Pulmonary Disease)	3 (17.6%)
Previous TB (Tuberculosis)	1 (5.9%)
Immunosuppression	3 (17.6%)
Surgical treatment for NTM-LD in the past	2 (11.8%)
**Symptoms at Diagnosis**	
Cough	16 (94.1%)
Hemoptysis	6 (35.3%)
Weight loss	6 (35.3%)
**NTM Species Identified**	
Mycobacterium avium	8 (47.0%)
Mycobacterium intracellulare	7 (41.2%)
*Mycobacterium abscessus* (subsp. abscessus)	6 (35.3%)
Combined infection	4 (23.5%)
**NTM Sensitivity**	
Macrolide sensitive	13 (76.4%)
Amikacin sensitive	15 (88.2%)
**Microbiological Diagnosis By**	
Sputum	7 (41.2%)
Bronchoscopy	12 (70.6%)
Both	2 (11.8%)
**Radiological Features**	
Extended Disease (>2 lobes)	17 (100%)
Bronchiectases	16 (94.1%)
Nodules	17 (100%)
Consolidation	8 (47.0%)
Cavity	3 (17.6%)

Abbreviations. NTM-LD: non-tuberculous mycobacterial lung disease, LTRIs: lower tract respiratory infections, COPD: chronic obstructive pulmonary disease, TB: tuberculosis.

**Table 2:** Treatment characteristics of patients with NTM-LD (*n* = 17).

Initial Regimen	No. of Patients (%)
**Drugs in the initial regimen**	
Azithromycin	12 (70.6%)
Clarithromycin	1 (5.9%)
Ethambutol	14 (82.3%)
Rifampicin	13 (76.5%)
Parenteral amikacin	13 (76.5%)
Linezolide	5 (29.4%)
Clofazimine	6 (35.3%)
Levofloxacin	4 (23.5%)
Trimethoprime–Sulfamethoxazole	1 (5.9%)
Tigecycline	6 (35.3%)
**Adverse effects**	
Ototoxicity	5 (29.4%)
Ocular toxicity	1 (5.9%)
Nephrotoxicity	3 (17.6%)
Hematocrit decrease	2 (11.8%)
**Regimens with ALIS**	**No. of Patients (%)**
**Reason for ALIS introduction**	
Refractoriness	5 (29.4%)
Recurrence	5 (29.4%)
Adverse effects to initial treatment	7 (41.1%)
**Co-administered drugs**	
Azithromycin	15 (88.2%)
Ethambutol	11 (64.7%)
Rifampicin	4 (23.5%)
Levofloxacin	3 (17.6%)
Linezolide	7 (41.2%)
Clofazimine	14 (82.3%)
Bedaquilline	11 (64.7%)
Trimethoprime–Sulfamethoxazole	1 (5.9%)
**Adverse effects**	
Hoarseness	6 (35.3%)
Dysphonia	5 (29.4%)
Bronchospasm	4 (23.5%)
Hypersensitivity pneumonitis	1 (5.9%)

Abbreviations. NTM-LD: non-tuberculous mycobacterial lung disease, ALIS: amikacin liposomal inhalation suspension.

## Data Availability

The data presented in this study are available on request from the corresponding author due to privacy reasons.
